# Antimalarial efficacy and toxicological assessment of medicinal plant ingredients of Prabchompoothaweep remedy as a candidate for antimalarial drug development

**DOI:** 10.1186/s12906-023-03835-x

**Published:** 2023-01-18

**Authors:** Prapaporn Chaniad, Tachpon Techarang, Arisara Phuwajaroanpong, Walaiporn Plirat, Parnpen Viriyavejakul, Abdi Wira Septama, Chuchard Punsawad

**Affiliations:** 1 Department of Medical Science, School of Medicine, Walailak University, Nakhon Si Thammarat, Thailand; 2 Research Center in Tropical Pathobiology, Walailak University, Nakhon Si Thammarat, 80160 Thailand; 3 Department of Tropical Pathology, Faculty of Tropical Medicine, Mahidol University, Bangkok, 10400 Thailand; 4 Research Center for Pharmaceutical Ingredient and Traditional Medicine, National Research and Innovation Agency (BRIN), Cibinong Science Center, Cibinong, West Java 16915 Indonesia

**Keywords:** Malaria, Antimalarial activity, Prabchompoothaweep remedy, *Terminalia arjuna*, Toxicity, GC-MS

## Abstract

**Background:**

Drug resistance exists in almost all antimalarial drugs currently in use, leading to an urgent need to identify new antimalarial drugs. Medicinal plant use is an alternative approach to antimalarial chemotherapy. This study aimed to explore potent medicinal plants from Prabchompoothaweep remedy for antimalarial drug development.

**Methods:**

Forty-eight crude extracts from Prabchompoothaweep remedy and its 23 plants ingredients were investigated in vitro for antimalarial properties using *Plasmodium* lactate dehydrogenase (pLDH) enzyme against *Plasmodium falciparum* K1 strain and toxicity effects were evaluated in Vero cells. The plant with promising antimalarial activity was further investigated using gas chromatography-mass spectrometry (GC-MS) to identify phytochemicals. Antimalarial activity in mice was evaluated using a four-day suppressive test against *Plasmodium berghei* ANKA at dose of 200, 400, and 600 mg/kg body weight, and acute toxicity was analyzed.

**Results:**

Of the 48 crude extracts, 13 (27.08%) showed high antimalarial activity against the K1 strain of *P. falciparum* (IC_50_ <  10 μg/ml) and 9 extracts (18.75%) were moderately active (IC_50_ = 11–50 μg/ml). Additionally, the ethanolic extract of Prabchompoothaweep remedy showed moderate antimalarial activity against the K1 strain of *P. falciparum* (IC_50_ = 14.13 μg/ml). Based on in vitro antimalarial and toxicity results, antimalarial activity of the aqueous fruit extract of *Terminalia arjuna* (IC_50_ = 4.05 μg/ml and CC_50_ = 219.6 μg/ml) was further studied in mice. GC-MS analysis of *T. arjuna* extract identified 22 compounds. The most abundant compounds were pyrogallol, gallic acid, shikimic acid, oleamide, 5-hydroxymethylfurfural, 1,1-diethoxy-ethane, quinic acid, and furfural. Analysis of the four-day suppressive test indicated that *T. arjuna* extract at dose of 200, 400, and 600 mg/kg body weight significantly suppressed the *Plasmodium* parasites by 28.33, 45.77, and 67.95%, respectively. In the acute toxicity study, *T. arjuna* extract was non-toxic at 2000 mg/kg body weight.

**Conclusions:**

The aqueous fruit extract of *T. arjuna* exerts antimalarial activity against *Plasmodium* parasites found in humans (*P. falciparum* K1) and mice (*P. berghei* ANKA). Acute toxicity studies showed that *T. arjuna* extract did not show any lethality or adverse effects up to a dose of 2000 mg/kg.

## Background

Malaria is an infectious disease transmitted to humans through the bites of infected female *Anopheles* mosquitoes. Five species of *Plasmodium* parasites, *Plasmodium falciparum*, *Plasmodium malariae*, *Plasmodium vivax*, *Plasmodium ovale*, and *Plasmodium knowlesi* cause malaria in humans [1]. The World Health Organization reported an estimated 241 million malaria cases worldwide, with an estimated 627,000 malaria deaths in 2020. The number of malaria cases in 2020 increased by 14 million compared with those in 2019 with accounted for 227 million cases [2]. Young children (under five years old), pregnant women, and non-immune people are the most vulnerable groups affected by malaria [2]. Antimalarial drug resistance has emerged as an essential problem requiring control. The first-line treatment, artemisinin-based combination therapy (ACTs) is now emergence and spread of resistance in the Greater Mekong subregion (GMS), which consists of Cambodia, Thailand, Vietnam, Myanmar and Laos. Resistance to the partner drugs piperaquine and mefloquine is also now common in the GMS, led to high failure rates of ACTs treatment [3, 4]. Therefore, there is an urgent need to develop novel therapeutic agents for malaria treatment.

Prabchompoothaweep remedy has been used in traditional Thai medicine for many years to treat allergic rhinitis and upper respiratory tract diseases [5, 6]. This remedy consists of 23 medicinal plants documented in the National List of Essential Medicine of Thailand. Among the medicinal plants, *Terminalia arjuna* Wight and Arn, commonly known as “arjuna” or “Sa-mor-tes” in Thai, has traditionally been used to treat several human diseases, including cardiovascular disorders (ischemia, cardiomyopathy, atherosclerosis, and myocardial necrosis) and blood diseases (anemia, and venereal and viral diseases) [7–9]. *T. arjuna* possesses several medicinal properties, including hypocholesterolemic, antibacterial, antimicrobial, antitumoral, antioxidant, anti-allergic, antifeedant, antifertility, and anti-human immunodeficiency virus activities [10–12].

However, the antimalarial properties and toxicity of Prabchompoothaweep remedy have not yet been reported. Therefore, this study aimed to investigate the in vitro antimalarial activity and toxicity of this remedy and its 23 medicinal plant ingredients. Additionally, a good candidate plant for antimalarial drug development was selected for further in vivo study of antimalarial activity and acute toxicity in mice.

## Methods

### Plant materials

Twenty-three plant ingredients from the Prabchompoothaweep remedy were purchased from a traditional Thai drug store in the Nakhon Si Thammarat Province, Thailand (Table 1). The use of plant materials complied with the relevant guidelines and regulations of the Plant Varieties Protection, Department of Agriculture, Ministry of Agriculture and Cooperatives, Thailand. The plant species were identified by Assoc. Prof. Tanomjit Supavita, School of Pharmacy at Walailak University. Voucher herbarium specimens were deposited in the School of Medicine, Walailak University (Table 1).

**Table 1:** List of plant materials used in the study

No	Plant species	Family	Common name	Plant part	Voucher number
1	*Acanthus ebracteatus*	Acanthaceae	Sea holly	Whole plant	SMD289001001
2	*Piper nigrum* L.	Piperaceae	Pepper	Fruit	SMD209001014
3	*Leonurus sibiricus*	Lamiaceae	Motherworth	Leaf	SMD142018001
4	*Kleinhovia hospital* L.	Sterculiaceae	Guest tree	Whole plant	SMD256009001
5	*Syzygium aromaticum* (L.) Merr. Et L.M. Perry.	Myrtaceae	Clove	Flower	SMD179013008
6	*Amorphophallus paeoniifolius* (Dennst.) Nicolson.	Araceae	Konjac	Whole plant	SMD019004032
7	*Terminalia arjuna* Wight and Arn.	Combretaceae	Arjuna	Fruit	SMD070006002
8	*Terminalia chebula* Retz.	Combretaceae	Chebulic myrobalans	Fruit	SMD070006007
9	*Plumbago indica* L.	Plumbaginaceae	Rosy leadwort	Roots	SMD212004002
10	*Zingiber officinale* Roscoe	Zingiberaceae	Ginger	Rhizomes	SMD288015005
11	*Lepidium sativum* L.	Cruciferae	Garden cress	Fruit	SMD079003001
12	*Anethum graveolens* L.	Apiaceae	Dill	Fruit	SMD276001001
13	*Foeniculum vulgare* Miller subsp. Var. vulgare	Umbelliferae	Sweet fennel	Fruit	SMD276010001
14	*Nigella sativa* L.	Ranunculaceae	Nigella	Seed	SMD228005001
15	*Angelica dahurica* Benth.	Umbelliferae	Dahurian angelica	Root	SMD276002003
16	*Atractylodes lancea* (Thung.) DC.	Asteraceae	Atractylodes	Root	SMD072010001
17	*Ardisia elliptica* Thunb.	Myrsinaceae	Shoebutton	Fruit	SMD178002017
18	*Enhalus acoroides* Zolls	Hydrocharitaceae	Tape Seagrass	Fruit	SMD132002001
19	*Piper chaba* Hunt	Piperaceae	Long pepper	Flower	SMD209002003
20	*Myristica fragrans* Houtt.	Myristicaceae	Nutmeg	Seed	SMD177004003–1
21	*Myristica fragrans* Houtt.	Myristicaceae	Nutmeg	Aril	SMD177004003–2
22	*Amomum testaceum* Ridl.	Zingiberaceae	Clustered cardamom	Fruit	SMD288002003
23	*Cinnamomum camphora* (L.) J. Presl.	Lauraceae	Camphor	Leaf	SMD143005003

### Plant extraction

All plant samples were cleaned with distilled water to remove dirt and dried at 60 °C in a hot air oven for 72 h. The plant samples were then cut into small pieces and weighed into portions of 60 g. Each plant was extracted using ethanol and distilled water. Ethanol was selected as a solvent due to it can dissolve most slightly non-polar and slightly polar molecules. Distilled water was used as the solvent to be related to the usage almost plants as Thai folk medicines. For ethanolic extraction, the plant samples (60 g) were macerated in 600 ml of 80% ethanol at 25–30 °C for 72 h and this procedure was repeated three times. The aqueous extract was obtained using the decoction method and 60 g of each plant was extracted three times by mixing with 600 ml of distilled water and boiled at 90–100 °C for 30 min. The resulting extract in each method was filtered through Whatman No. 1 filter paper, evaporated in a rotary evaporator (N-1200B, EYELA Co., Ltd., Shanghai, China) at 60 °C, and lyophilized to dryness using a freeze-dryer (Gamma 2–16 LSCplus, Martin Christ, Osterode am Harz, Germany). The crude extracts were collected and stored at 4 °C until use.

### Phytochemical analysis

All the extracts were subjected to standard phytochemical analyses to determine the presence of flavonoids, terpenoids, alkaloids, tannins, anthraquinone, cardiac glycosides, saponins, and coumarins, as previously described with some modifications [13, 14].

### In vitro cultivation of *Plasmodium falciparum*

To investigate in vitro antimalarial activity, *P. falciparum* K1 strain was cultured as previously described with minor modifications [15]. The *Plasmodium* parasite was cultured in uninfected O^+^ red blood cells (RBCs) as host cells and maintained in complete medium (RPMI-1640) containing 2 mg/ml sodium bicarbonate, 10 μg/ml hypoxanthine (Sigma-Aldrich, New Delhi, India), 4.8 mg/ml HEPES (HiMedia, Mumbai, India), 0.5% Albumax II (Gibco, Waltham, MA, USA), and 2.5 μg/ml gentamicin (Sigma-Aldrich). The culture flasks were incubated at 37 °C and 5% CO_2_. The percentage of parasitemia was monitored daily using a light microscope.

### In vitro antimalarial activity assay

Prabchompoothaweep remedy and its plant ingredient extracts were tested for their antimalarial activity using an in vitro *Plasmodium* lactate dehydrogenase (pLDH) assay [16]. In this assay plates containing 2% parasite cultures were incubated with crude extract at final concentrations between 0.3–2500 mg/ml for 72 h at 37 °C in a CO_2_ incubator. Artesunate (0.3–2500 mg/ml) (Sigma-Aldrich) and dimethyl sulfoxide (DMSO; Merck, Darmstadt, Germany) were added to each well as positive and negative controls, respectively. Non-infected RBCs were used as blank controls. After 72 h of incubation, the plates were frozen at − 20 °C and thawed at 37 °C three times. The supernatant from each well was transferred to a new microplate containing the Malstat reagent (Sigma-Aldrich). Nitroblue tetrazolium/phenazine ethosulfate solution (Sigma-Aldrich) was added to the plate and cultured in the dark for 60 min. Next, 5% acetic acid (Merck) was added to each well to stop the reaction. The absorbance at 650 nm was measured using a microplate reader. Each sample was tested in triplicates. Finally, a log dose-response curve was generated and used to determine the percent inhibition and half-maximal inhibitory concentration (IC_50_).

### In vitro cytotoxicity assay

Vero cells (1 × 10^5^/well) were plated in 200 μl of complete medium per well in 96-well plates. After cell attachment, the plant extracts were added and incubated at 37 °C for 24 h. Concentrations of plant extract varied from 0 to 80 μg/ml. The culture medium was then replaced with 100 μl of fresh medium/well containing 3-(4, 5-dimethylthiazol-2-yl)-2, 5-diphenyltetrazolium bromide (MTT) per well and incubated at 37 °C for 3 h. DMSO was then added to each well and incubated for another 20 min at room temperature in the dark. Lastly, the absorbance at 560 and 670 nm was measured using a microplate reader. All experiments were repeated thrice. The 50% cytotoxic concentration (CC_50_) of the extracts was determined by dose-response curve analysis [17].

### Selectivity index

A selectivity index (SI), which is the ratio between cytotoxic and antimalarial activities [18], was calculated for each extract according to the following formula:

$$SI=CC_{50}/IC_{50}$$

### GC-MS analysis

The relative quantities of the phytochemicals present in the extracts were determined using gas chromatography with a 7000C Triple Quadrupole GC/MS (Agilent Technologies, Santa Clara, CA, USA) equipped with an HP-5MS column (30 m × 0.25 mm; 0.25 μm). Spectroscopic detection by GC-MS involves an electron ionization system that utilizes high energy electrons of 70 eV, ion source temperature of 250 °C, and mass scanning range of 33–600 amu in full scan. Pure helium gas (99.99%) was used as the carrier gas at a constant flow rate of 1 ml/min. The injector temperature was maintained at a constant of 250 °C, and the oven temperature was programmed as follows: 60 °C for 2 min, 150 °C at an increasing rate of 10 °C/min, and finally, 300 °C at an increasing rate of 5 °C/min. The sample (1 μl) in ethanol was injected in the split mode at a split ratio of 20:1, respectively. The compounds in the test samples were identified by comparing their retention times and mass spectra with those in the spectral database of the National Institute of Standards and Technology (NIST2011) structural library. Only peaks with 80% similarity and above with the NIST libraries were selected and identified.

### Animals

Male Institute of Cancer Research (ICR) mice aged 6–8 weeks old and weighing 25–30 g in body weight were purchased from Nomura Siam International Co., Ltd. (Pathumwan, Bangkok, Thailand). The mice housing temperature was maintained at a room temperature of approximately 22 °C (± 3 °C) and relative humidity of 50–60%. The lighting environment was set to a 12:12 h light/dark cycle. Mice were allowed free access to food pellets and clean drinking water.

### Four-day suppressive test (Peter’s test)

The four-day suppressive test was used to measure the schizonticidal activity of the aqueous extract of *T. arjuna* against *P. berghei* ANKA-infected ICR mice. The method was performed as previously described with minor modifications [19, 20]. Briefly, male ICR mice were randomly divided into five groups of five animals. Twenty-five mice were injected with 1 × 10^7^ RBCs infected with *P. berghei* ANKA via intraperitoneal injection [20]. The treatment started 4 h following inoculation. In the extract treatment groups, the animals received daily oral doses of 200, 400, or 600 mg/kg body weight aqueous extract of *T. arjuna* in 200 μl of 7% Tween 80 solution. The dosage was selected with increasing as low, moderate and high doses of crude extract with 200, 400, and 600 mg/kg body weight according to previous studies [20–22]. The negative control group received 200 μl of 7% Tween 80 solution, while the positive control group was administered 6 mg artesunate/kg body weight orally per day. The mice were administered each substance daily for 4 days (at 4, 24, 48, and 72 h after inoculation). On the fifth day, the percentage of parasitemia was determined using Giemsa staining. Percent inhibition was calculated using the following formula [23]:

$$\%\textrm{inhibition}=\frac{\textrm{Parasitemia}\ \left(\textrm{negative}\ \textrm{control}\right)-\textrm{Parasitemia}\ \left(\textrm{treated}\ \textrm{group}\right)\ }{\textrm{Parasitemia}\ \left(\textrm{negative}\ \textrm{control}\right)}\times 100$$

### Determination of mean survival time (MST)

MST was determined as described by Chaniad et al. [20]. Twenty-five mice were used in the four-day suppressive test and fed ad libitum. Mouse mortality was monitored daily until day 30 after parasite inoculation. Any deaths in the treatment and control groups that occurred during the follow-up period were recorded. The MST for each group was calculated using the following formula [23]:

$$\textrm{MST}=\kern2.5em \frac{\textrm{Sum}\ \textrm{of}\ \textrm{survival}\ \textrm{time}\ \textrm{of}\ \textrm{all}\ \textrm{mice}\ \textrm{in}\ \textrm{each}\ \textrm{group}\ \left(\textrm{days}\right)}{\textrm{Total}\ \textrm{number}\ \textrm{of}\ \textrm{mice}\ \textrm{in}\ \textrm{given}\ \textrm{group}}\times 100$$

### Acute toxicity test

The crude aqueous extract of *T. arjuna* was assessed for toxicity in non-infected ICR mice aged 6–8 weeks old and weighing 25–30 g according to the standard guidelines of the Organization for Economic Cooperation and Development [24]. Fifteen mice were randomly divided into three groups of five mice each: mice treated with 2000 mg/kg *T. arjuna* aqueous extract, negative control, and untreated. The aqueous extract of *T. arjuna* was dissolved in 7% Tween 80 to a dose of 2000 mg/kg body weight. Mice in the experimental group received a single dose of 2000 mg/kg *T. arjuna* extract orally, while mice in the control group were administered 200 μl of 7% Tween 80 solution. Blood samples were collected into heparinized tubes using a cardiac puncture technique. The plasma samples were used for biochemical analysis of liver function (alanine aminotransferase [ALT], aspartate aminotransferase [AST], and alkaline phosphatase [ALP]) and kidney function (blood urea nitrogen [BUN] and creatinine [Cr]) using an AU480 chemistry analyzer (Beckman Coulter, Brea, CA, USA). Furthermore, liver and kidney tissues were removed and fixed in formalin for histopathological examination.

### Histopathology

Histopathological examination of the liver and kidney tissues was performed according to previously described histological procedures [25, 26]. All tissue were fixed in 10% buffered formalin, then dehydrated using a gradient series of ethanol solutions, rinsed three times with xylene, and placed in a mold containing paraffin. The paraffin blocks were then serially sectioned at 5 μm thickness, transferred to glass slides, and stained with hematoxylin and eosin solution. To evaluate histopathological changes, the stained slides were observed using a light microscope by two independent researchers blinded to the experimental groups.

### Statistical analysis

The results are presented as mean ± standard error of the mean (SEM). IBM SPSS Statistics version 23.0 software was used for the statistical analysis. The Kolmogorov-Smirnov goodness-of-fit test was used to test the normal distribution. The statistical significance of parasitemia inhibition was analyzed using one-way analysis of variance, followed by Tukey’s multiple comparison test. Statistical significance was set at *p*-value less than 0.05 (*p* ≤ 0.05).

## Results

### Extraction of plant materials

The percentage of crude extract yield (%yield) as shown in Table 2. Extraction of the roots of *A. lancea* with water produced the highest crude extract, 31.60 g of dark brown solid were afforded after freeze drying representing 52.67% yield with respect to the plant material used. Ethanolic extract was produced with brown sticky mixed with yellow liquid with 13.26 g accounting for a 22.11% yield. Extraction of the fruits of *T. arjuna* with ethanol, 8.85 g of a caramel sticky solid were obtained representing 14.76% yield and aqueous extract was afforded as 27.87 g of caramel sticky solid translating to 46.44% yield. Preparation of *T. chebula* extracts in ethanol produced dark brown semi-solid with the 25.58% yield and aqueous extract also produced dark brown semi-solid with 36.15% yield.

**Table 2:** Extraction yields and colour and appearance of ethanolic and aqueous extracts of the medicinal plants in Prabchompoothaweep remedy

Plant species	Ethanolic extract		Aqueous extract	
	Yield (% w/w)	Colour and appearance	Yield (% w/w)	Colour and appearance
*A. ebracteatus*	5.23	black solid mixed with brown liquid	10.66	dark brown crumbly solid
*P. nigrum*	7.25	brown semi-solid	32.80	brown crumbly solid
*L. sibiricus*	3.12	black sticky solid	25.27	black sticky solid
*K. hospital*	0.53	brown sticky solid	2.94	brown, less sticky solid
*S. aromaticum*	22.43	brown sticky solid	28.10	copper crumbly solid
*A. paeoniifolius*	10.97	light brown crumbly solid	20.69	beige velvet solid
*T. arjuna*	14.76	caramel sticky solid	46.44	caramel sticky solid
*T. chebula*	25.58	dark brown semi-solid	36.15	dark brown semi-solid
*P. indica*	5.59	black less sticky solid	29.31	dark brown crumbly solid
*Z. officinale*	1.00	red brown less sticky solid	10.35	dark brown crumbly solid
*L. sativum*	0.55	brown sticky solid	11.03	beige velvet solid
*A. graveolens*	3.17	dark brown solid mixed with brown liquid	15.81	brown crumbly solid
*F. vulgare*	3.91	dark brown solid mixed with brown liquid	22.39	brown sticky solid
*N. sativa*	0.52	dark green sticky solid	11.94	black sticky solid
*A. dahurica*	4.54	dark brown sticky solid	26.86	light brown velvet solid
*A. lancea*	22.11	brown sticky mixed with yellow liquid	52.67	dark brown solid
*A. elliptica*	9.71	black sticky solid	22.04	radish brown crumbly solid
*E. acoroides*	2.59	black crumbly solid	14.50	dark gray crumbly solid
*P. chaba*	11.54	orange creamy	17.27	red brown solid
*M. fragrans* (seed)	12.17	orange wax	13.75	light brown velvet
*M. fragrans* (aril)	20.71	orange sticky mixed with orange liquid	31.19	orange color with spongy solid
*A. testaceum*	0.97	light brown sticky	8.14	dark brown crumbly solid
*C. camphora*	2.14	light brown sticky	3.31	dark brown sticky
Remedy	5.43	dark green sticky	27.78	dark brown crumbly solid

### Phytochemical screening

Phytochemical analysis of each plant component in Prabchompoothaweep remedy revealed the presence of flavonoids, terpenoids, alkaloids, tannins, saponins, and coumarins, whereas anthraquinones and cardiac glycosides were not detected in any of the extracts (Table 3). Moreover, the ethanolic extract of the remedy contained terpenoids, alkaloids, tannins, and coumarins, whereas the aqueous extract of the remedy contained flavonoids, terpenoids, alkaloids, tannins, saponins, and coumarins (Table 3).

**Table 3:** Phytochemical constituents of ethanolic and aqueous extracts of the medicinal plants in Prabchompoothaweep remedy

Plant species	Extract	Phytochemical constituents							
		FL	TN	AL	TA	AN	CG	SA	CM
*A. ebracteatus*	Ethanolic	**–**	**+**	**+**	**–**	**–**	**–**	**–**	**–**
	Aqueous	**+**	**+**	**+**	**+**	**–**	**–**	**+**	**–**
*P. nigrum*	Ethanolic	**+**	**–**	**++**	**–**	**–**	**–**	**–**	**+**
	Aqueous	**+**	**+**	**–**	**–**	**–**	**–**	**+**	**–**
*L. sibiricus*	Ethanolic	**–**	**+**	**+++**	**–**	**–**	**–**	**–**	**–**
	Aqueous	**+**	**–**	**+++**	**–**	**–**	**–**	**+**	**–**
*K. hospital*	Ethanolic	**+**	**–**	**++**	**–**	**–**	**–**	**–**	**–**
	Aqueous	**+**	**–**	**++**	**–**	**–**	**–**	**++**	**–**
*S. aromaticum*	Ethanolic	**+**	**+**	**+**	**+++**	**–**	**–**	**+**	**–**
	Aqueous	**+**	**+**	**+**	**+++**	**–**	**–**	**+++**	**–**
*A. paeoniifolius*	Ethanolic	**–**	**+**	**+**	**–**	**–**	**–**	**–**	**–**
	Aqueous	**–**	**+**	**–**	**–**	**–**	**–**	**+++**	**–**
*T. arjuna*	Ethanolic	**+**	**–**	**+**	**++**	**–**	**–**	**+**+	**–**
	Aqueous	**+**	**+**	**+**	**++**	**–**	**–**	**+**	**–**
*T. chebula*	Ethanolic	**+**	**–**	**+**	**+++**	**–**	**–**	**++**	**–**
	Aqueous	**+**	**–**	**+**	**+++**	**–**	**–**	**++**	**–**
*P. indica*	Ethanolic	**+**	**+**	**+**	**–**	**–**	**–**	**–**	**–**
	Aqueous	**+**	**–**	**+**	**++**	**–**	**–**	**+++**	**–**
*Z. officinale*	Ethanolic	**+**	**+**	**+**	**–**	**–**	**–**	**–**	**+**
	Aqueous	**+**	**–**	**–**	**–**	**–**	**–**	**+++**	**–**
*L. sativum*	Ethanolic	**+**	**+**	**++**	**–**	**–**	**–**	**–**	**+**
	Aqueous	**–**	**–**	**++**	**–**	**–**	**–**	**+**	**+**
*A. graveolens*	Ethanolic	**+**	**+**	**+++**	**–**	**–**	**–**	**–**	**–**
	Aqueous	**+**	**–**	**+**	**+**	**–**	**–**	**–**	**–**
*F. vulgare*	Ethanolic	**+**	**+**	**+**	**–**	**–**	**–**	**–**	**+**
	Aqueous	**+**	**+**	**+**	**+**	**–**	**–**	**–**	**–**
*N. sativa*	Ethanolic	**–**	**+**	**+**	**–**	**–**	**–**	**–**	**–**
	Aqueous	**+**	**+**	**+**	**+**	**–**	**–**	**+++**	**–**
*A. dahurica*	Ethanolic	**+**	**+**	**+**	**–**	**–**	**–**	**–**	**+**
	Aqueous	**+**	**+**	**–**	**–**	**–**	**–**	**–**	**–**
*A. lancea*	Ethanolic	**–**	**+**	**+**	**–**	**–**	**–**	**–**	**–**
	Aqueous	**+**	**+**	**+**	**–**	**–**	**–**	**–**	**–**
*A. elliptica*	Ethanolic	**–**	**–**	**+**	**+**	**–**	**–**	**–**	**–**
	Aqueous	**+**	**–**	**+**	**++**	**–**	**–**	**++**	**–**
*E. acoroides*	Ethanolic	**–**	**+**	**+**	**–**	**–**	**–**	**–**	**–**
	Aqueous	**+**	**+**	**–**	**–**	**–**	**–**	**–**	**–**
*P. chaba*	Ethanolic	**+**	**+**	**++**	**–**	**–**	**–**	**–**	**+**
	Aqueous	**+**	**+**	**–**	**–**	**–**	**–**	**+**	**+**
*M. fragrans* (seed)	Ethanolic	**–**	**+**	**+**	**–**	**–**	**–**	**–**	**+**
	Aqueous	**–**	**+**	**–**	**–**	**–**	**–**	**–**	**–**
*M. fragrans* (aril)	Ethanolic	**+**	**+**	**+**	**–**	**–**	**–**	**–**	**+**
	Aqueous	**–**	**+**	**–**	**–**	**–**	**–**	**–**	**–**
*A. testaceum*	Ethanolic	**+**	**+**	**+**	**–**	**–**	**–**	**–**	**+**
	Aqueous	**+**	**+**	**+**	**–**	**–**	**–**	**+**	**–**
*C. camphora*	Ethanolic	**–**	**++**	**+**	**+**	**–**	**–**	**–**	**–**
	Aqueous	**–**	**++**	**+**	**+**	**–**	**–**	**–**	**–**
Remedy	Ethanolic	**–**	**+**	**++**	**++**	**–**	**–**	**–**	**+**
	Aqueous	**+**	**+**	**+**	**++**	**–**	**–**	**+**	**+**

*FL* Flavonoids, *TN* Terpenoids, *AL* Alkaloids, *TA* Tannins, *AN* Anthraquinones, *CG* Cardiac glycosides, *SA* Saponins, *CM* Coumarins

+++: high presence; ++: moderate presence; +: low presence; −: absence

### In vitro antimalarial activity

The in vitro antimalarial activity of Prabchompoothaweep remedy and its ingredients is shown in Table 4. The activity of the extracts was considered high if IC_50_ <  10 μg/ml, moderately active if IC_50_ ranged between 11 and 50 μg/ml, mildly active if IC_50_ ranged between 51 and 100 μg/ml, and inactive if IC_50_ >  100 μg/ml [27]. According to these criteria, 13 extracts (27.08%) of 10 plants showed high antimalarial activity against the K1 strain of *P. falciparum* with IC_50_ values lower than 10 μg/ml. Nine extracts (18.75%) were moderately active and five extracts (10.42%) possessed mild activity. Of the total tested plant extracts, the aqueous flower extract of *S. aromaticum* was the most active against *P. falciparum*, with the lowest IC_50_ value (1.96 μg/ml), followed by the ethanolic flower extract of *P. chaba*, ethanolic rhizome extract of *Z. officinale*, aqueous fruit extract of *T. arjuna*, ethanolic fruit extract of *P. nigrum*, and ethanolic fruit extract of *T. arjuna* (IC_50_ = 2.06, 3.42, 4.05, 4.38, and 4.72 μg/ml, respectively). The ethanolic extract of Prabchompoothaweep showed moderate antimalarial activity against the K1 strain of *P. falciparum* (IC_50_ = 14.13 μg/ml). Artesunate, the positive control, exhibited antimalarial activity at an IC_50_ of 1.25 ng/ml.

**Table 4:** In vitro antimalarial activity and cytotoxicity of ethanolic and aqueous extracts of Prabchompoothaweep remedy ingredients

No	Plant species	Part used	Ethanolic extract			Aqueous extract		
			IC_**50**_ (μg/ml)	CC_**50**_ (μg/ml)	SI	IC_**50**_ (μg/ml)	CC_**50**_ (μg/ml)	SI
1	*A. ebracteatus*	Whole plant	18.94 ± 4.82	96.43 ± 14.32	5.09	> 200	114.00 ± 14.10	< 0.57
2	*P. nigrum*	Fruit	4.38 ± 2.58	194.30 ± 61.00	44.36	> 200	185.40 ± 0.40	< 0.93
3	*L. sibiricus*	Leaf	13.65 ± 0.35	20.51 ± 0.15	1.50	> 200	185.70 ± 2.20	< 0.93
4	*K. hospital*	Whole plant	14.39 ± 12.99	111.00 ± 7.95	7.71	77.36 ± 6.34	> 200	2.81
5	*S. aromaticum*	Flower	7.15 ± 6.79	> 200	> 27.97	1.96 ± 0.88	134.70 ± 14.09	68.72
6	*A. paeoniifolius*	Whole plant	152.93 ± 0.68	140.50 ± 1.42	0.92	> 200	> 200	> 1.00
7	*T. arjuna*	Fruit	4.72 ± 2.28	> 200	> 42.37	4.05 ± 0.54	> 200	> 54.22
8	*T. chebula*	Fruit	5.05 ± 1.86	199.30 ± 40.45	39.47	4.53 ± 0.82	> 200	> 106.78
9	*P. indica*	Roots	8.43 ± 5.27	> 200	> 23.72	> 200	> 200	> 1.00
10	*Z. officinale*	Rhizomes	3.42 ± 1.68	105.10 ± 13.85	30.73	56.96 ± 0.65	136.80 ± 8.90	2.40
11	*L. sativum*	Fruit	68.80 ± 3.54	57.53 ± 1.365	0.84	> 200	145.40 ± 15.45	< 0.73
12	*A. graveolens*	Fruit	27.28 ± 0.94	49.51 ± 1.54	1.81	> 200	> 200	> 1.00
13	*F. vulgare*	Fruit	16.04 ± 1.53	31.50 ± 1.40	1.96	157.77 ± 1.00	96.23 ± 34.55	0.61
14	*N. sativa*	Seed	57.32 ± 1.40	88.46 ± 23.96	1.54	> 200	> 200	< 6.55
15	*A. dahurica*	Root	28.10 ± 0.82	69.92 ± 8.77	2.49	146.70 ± 1.41	> 200	> 1.36
16	*A. lancea*	Root	7.37 ± 7.72	29.54 ± 0.63	4.01	> 200	167.30 ± 7.05	< 0.84
17	*A. elliptica*	Fruit	7.08 ± 0.72	113.30 ± 0.45	16.00	126.98 ± 5.12	> 200	> 1.58
18	*E. acoroides*	Fruit	> 200	93.74 ± 8.66	< 0.47	> 200	146.30 ± 7.90	< 0.73
19	*P. chaba*	Flower	2.06 ± 0.62	198.60 ± 7.9	96.41	> 200	> 200	< 1.44
20	*M. fragrans*	Seed	13.68 ± 0.54	> 200	> 14.62	> 200	77.09 ± 4.43	< 0.39
21	*M. fragrans*	Aril	5.96 ± 1.73	38.30 ± 7.71	6.43	> 200	113.80 ± 17.71	< 0.57
22	*A. testaceum*	Fruit	13.76 ± 0.59	132.90 ± 12.75	9.66	144.77 ± 0.50	> 200	> 1.38
23	*C. camphora*	Leaf	59.65 ± 1.02	> 200	> 3.35	62.21 ± 2.01	> 200	> 3.21
24	Remedy		14.13 ± 0.42	121.60 ± 17.9	8.61	137.10 ± 2.75	161.8 ± 72.12	1.18
	Artesunate	IC_50_ = 1.25 ± 0.52 ng/ml						
	Doxorubicin	CC_50_ = 1.60 ± 0.23 μg/ml						

Data are presented as the mean ± standard error of the mean (SEM)

IC_50_: 50% inhibition concentration; CC_50_: 50% cytotoxic concentration

### In vitro cytotoxicity

The evaluation of in vitro toxicity in Vero cells is shown in Table 4. A non-toxic effect is defined as a CC_50_ value greater than 50 μg/ml [28]. Therefore, all extracts were non-toxic to Vero cells with CC_50_ values greater than 50 μg/ml, except five ethanolic extracts that showed toxic effects: *L. sibiricus* leaf*, A. lancea* root, *F. vulgare* fruit, *M. fragrans* aril, and *A. graveolens* fruit (CC_50_ = 20.51, 29.54, 31.50, 38.30, and 49.51 μg/ml, respectively).

Of the total 48 extracts, the aqueous extract of *T. arjuna* was found in the top five extracts with an antimalarial effect and was non-toxic to Vero cells. This extract showed promising antimalarial activity (IC_50_ = 4.05 μg/ml) against the K1 strain of *P. falciparum* and no cytotoxic effect against Vero cells (CC_50_ >  200 μg/ml). Based on the high antimalarial activity and SI values obtained for the aqueous fruit extract of *T. arjuna* and no previous report of its antimalarial activity, the in vivo antimalarial activity and acute toxicity of this extract was further evaluated in mice.

### GC-MS analysis of ethanolic aqueous fruit extract of *T. arjuna*

**Fig. 1:**
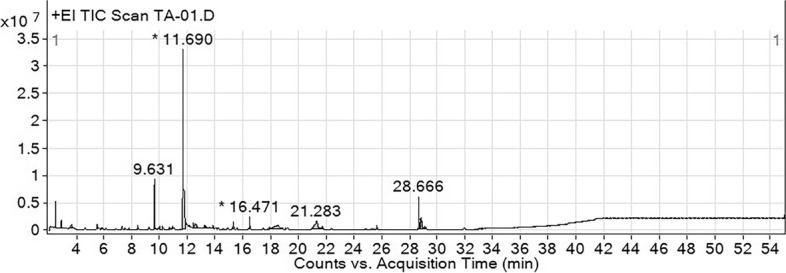
GC-MS chromatogram of the aqueous fruit extract of *T. arjuna*

**Fig. 2:**
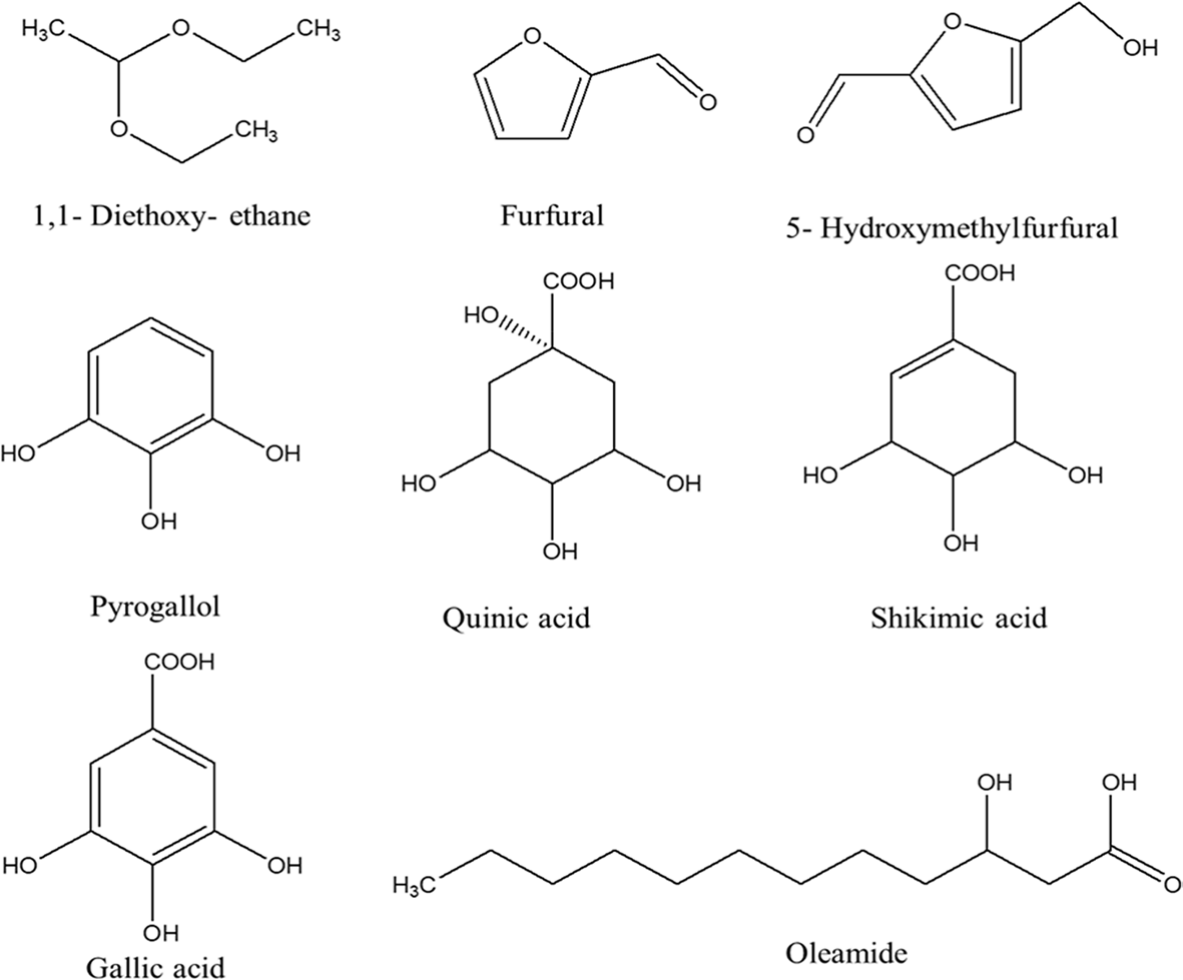
Chemical structure of eight compounds of aqueous *T. arjuna* extract identified by GC-MS with the peak area greater than 1%

**Fig. 3:**
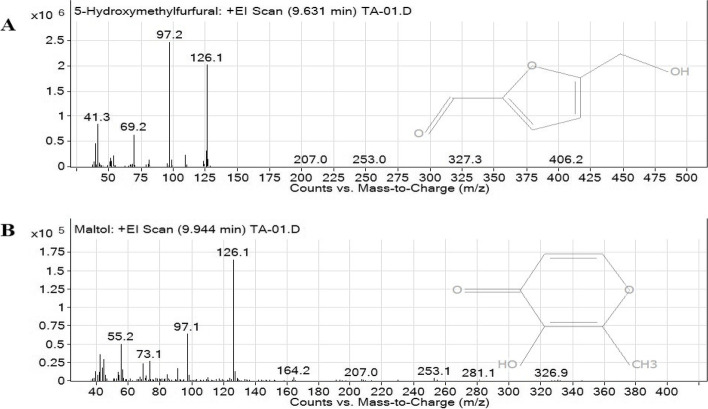
The mass spectrum of 5-hydroxymethylfurfural (C_6_H_6_O_3_; **A**), and maltol (C_6_H_6_O_3_; **B**)

**Fig. 4:**
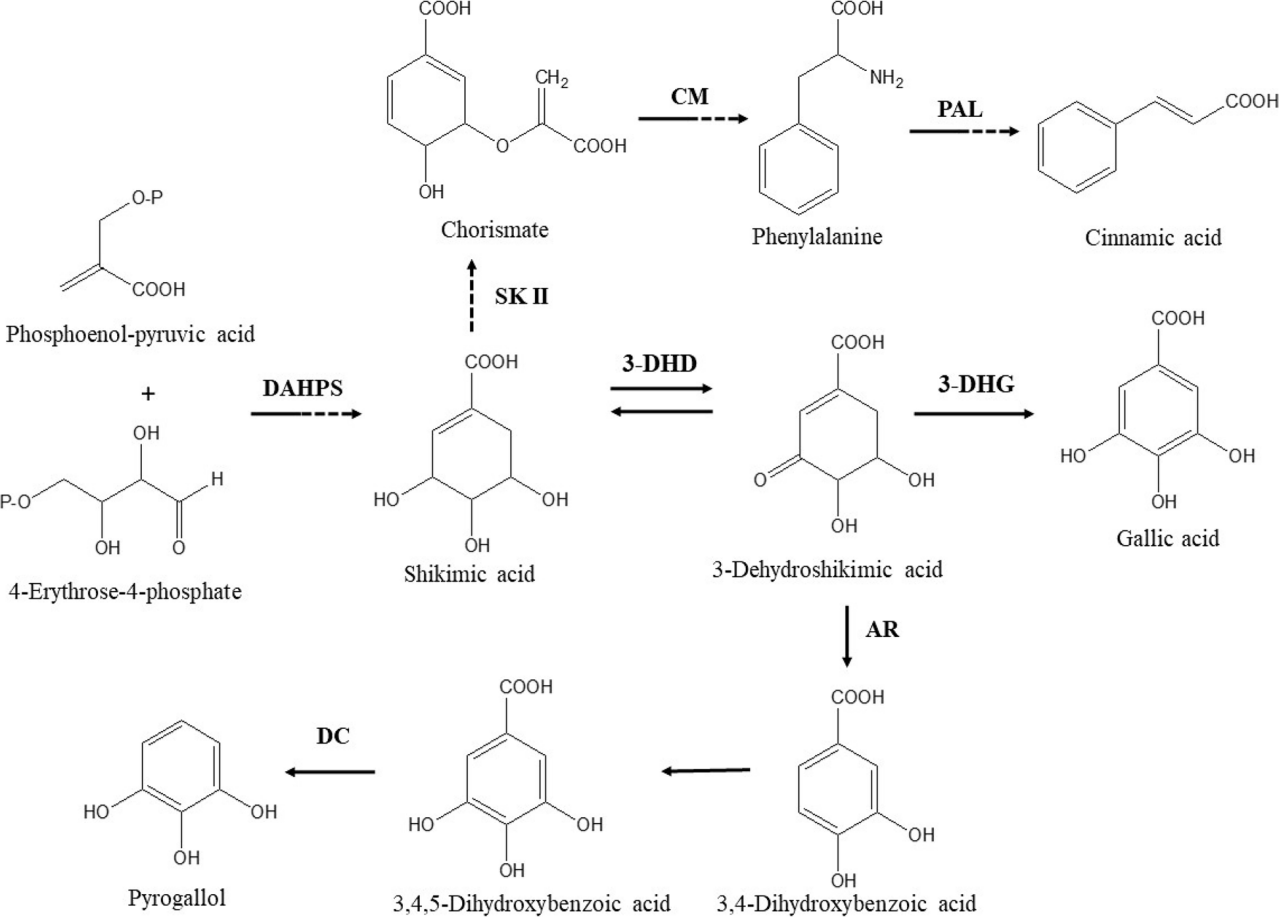
Biosynthesis pathay of shikimic cid, cinnamic acid, gallic acid and pyrogallol. DAHPS: 3-deoxy-D-arabino-heptulosonate-7-phosphate synthase; SK II: shikimate kinase II; CM: chorismate mutase; PAL: phenylalanine ammonia lyase; 3-DHD: 3-dehydroshikimate dehydratase; 3-DHG: 3-dehydroshikimate dehydrogenase; AR: aromatase; DC: decarboxylase [29–31].

The GC-MS chromatograms of the fruit extract of *T. arjuna* are shown in Fig. 1. The mass spectra of the phytochemical compounds were compared with those in the spectral database of known compounds in the NIST library. Twenty-two compounds were identified and characterized (Table 5)**.** The most abundant compound was pyrogallol with a retention time of 11.690 min (40.69%), followed by gallic acid (9.87%), shikimic acid (7.19%), oleamide (6.11%), and 5-hydroxymethylfurfural (5.72%), 1,1-diethoxy-ethane (3.11%), quinic acid (2.44%) and furfural (1.08%). Other compounds were present at concentrations below 1%. Chemical structure of eight compounds with the peak area greater than 1% was illustrated in Fig.2. Particularly, 5-hydroxymethylfurfural, compounds 8 with a retention time of 9.631 and maltol, compound 9 with a retention time of 9.944 have the same formula as C_6_H_6_O_3_ but the spectrum patterns are different (Fig. 3). Interestingly, the identified compounds, i.e., benzenetriol (pyrogallol), trihydroxybenzoic acid (gallic acid), shikimic acid, and cinnamic acid, are interrelated via the biosynthesis pathway (Fig.4).

**Table 5:** Compounds identified in the aqueous fruit extract of *T. arjuna* by GC-MS

Peak	Retention time (min)	Name of the compounds	Molecular formula	Molecular weight	Peak area (%)
1	2.472	1,1-Diethoxy-ethane	C_6_H_14_O_2_	118	3.11
2	2.910	2-Hydroxypropanenitrile	C_3_H_5_NO	71	0.76
3	3.621	Furfural	C_5_H_4_O_2_	96	1.08
4	5.491	Glycerin	C_3_H_8_O_3_	92	0.81
5	5.920	Tetraethyl silicate	C_8_H_2_OO_4_Si	208	0.26
6	7.286	Maltol	C_6_H_6_O_3_	126	0.36
7	8.420	Pyranone	C_6_H_8_O_4_	144	0.58
8	9.631	5-Hydroxymethylfurfural	C_6_H_6_O_3_	126	5.72
9	9.944	Maltol	C_6_H_6_O_3_	126	0.28
10	10.222	Diethyl hydroxybutanoate	C_8_H_14_O_5_	190	0.46
11	11.690	Pyrogallol	C_6_H_6_O_3_	126	40.69
12	12.414	Cinnamic acid	C_9_H_8_O_2_	148	0.72
13	13.186	3-Carboxyphenol	C_7_H_6_O_3_	138	0.69
14	13.839	2,4-Di-tert-butylphenol	C_14_H_22_O	206	0.41
15	16.471	Quinic acid	C_7_H_12_O_6_	192	2.44
16	18.482	Shikimic acid	C_7_H_10_O_5_	174	7.19
17	21.283	Gallic acid	C_7_H_6_O_5_	170	9.87
18	22.389	Ethyl palmitate	C_18_H_36_O_2_	284	0.20
19	24.887	cis-Vaccenic acid	C_18_H_34_O_2_	282	0.18
20	25.463	Ethyl oleate	C_20_H_38_O_2_	310	0.23
21	25.623	Hexadecanamide	C_16_H_33_NO	255	0.83
22	28.666	Oleamide	C_18_H_35_NO	281	6.11

### Four-day suppressive test

The four-day suppressive test showed that mice treated with aqueous fruit extract of *T. arjuna* at concentrations of 200, 400, and 600 mg/kg presented significantly (*p* <  0.001) lower percentages of parasitemia (24.58, 18.60, and 10.99%, respectively) compared with that in the negative control group (34.30%). Mice treated with aqueous fruit extracts of *T. arjuna* exhibited parasite suppression rates in a dose-dependent manner, with a maximum activity of 67.95%, followed by 45.77 and 28.33% at doses of 600, 400 and 200 mg/ml, respectively (Table 6). The survival time of all mice was also assessed over a 30-d period, as shown in Table 6. The MST of the extract-treated groups was dose-dependent. Extract doses of 200, 400, and 600 mg/kg body weight significantly (*p* <  0.05) prolonged the survival time by 13.00, 16.00, and 17.40 d, respectively, compared with that in the negative control mice (8.60 d). Additionally, compared with the 200 mg/kg extract-treated group, the mean survival durations of the 400 and 600 mg/kg extract-treated groups were significantly extended (Table 6).

**Table 6:** In vivo antiplasmodial suppression of aqueous fruit extract of *T. arjuna* in ICR mice blood infected with *P. berghei* ANKA

Group	Dose (mg/ml)	% Parasitemia	% Suppression	MST (days)
Negative control	–	34.30 ± 2.52 ^b, c, d, e^	–	8.60 ± 1.03
Artesunate	6	2.38 ± 0.35 ^a, c, d, e^	93.07 ± 1.01 ^a, c, d, e^	18.40 ± 0.98 ^a, c, d, e^
*T. arjuna* extract (Treated groups)	200	24.58 ± 1.15 ^a, b, d, e^	28.33 ± 3.01 ^a, b, d, e^	13.00 ± 1.67 ^a, b, d, e^
	400	18.60 ± 1.64 ^a, b, c, e^	45.77 ± 4.78 ^a, b, c, e^	16.00 ± 1.05 ^a, b, c^
	600	10.99 ± 1.40 ^a, b, c, d^	67.95 ± 4.09 ^a, b, c, d^	17.40 ± 1.60 ^a, b, c^

Data represent mean ± SEM (*n* = 5 per group); MST: mean survival time; Negative control: 7% Tween 80 solution

Values are significantly different at *p* < 0.05

Significant differences (*p* < 0.01) are indicated by ^a^compared to the negative control; ^b^compared to artesunate; ^c^compared to 200 mg/kg extract; ^d^compared to 400 mg/kg extract; ^e^compared to 600 mg/kg extract

### In vivo acute toxicity biochemical tests

All mice treated with 2000 mg/kg aqueous fruit extract of *T. arjuna* revealed no gross physical or behavioral changes, including lacrimation, altered feeding activities, vomiting, diarrhea, abnormal secretion, abnormal sleep, excitement, and hair erection for 24 h, and no mortality occurred during the 14-d follow-up period. Therefore, the lethal dose of the extract was greater than 2000 mg/kg body weight. To determine the effects of the aqueous fruit extract of *T. arjuna* on the liver and kidney, plasma biomarkers of liver and kidney functions were examined. The findings demonstrated that the mean levels of ALT, ALP, BUN, and Cr in the mice treated with 2000 mg/kg *T. arjuna* extract did not significantly differ from those in the 7% Tween 80 and untreated control groups (Table 7). However, the mean levels of AST in mice treated with 2000 mg/kg *T. arjuna* extract were significantly higher than those in the 7% Tween 80 group (*p* <  0.05).

**Table 7:** Plasma biomarkers of liver and kidney function after 2000 mg/kg aqueous *T. arjuna* extract treatment

Group	Liver function test		
	AST (U/L)	ALT (U/L)	ALP (U/L)
Untreated control	85.42 ± 7.04	42.65 ± 4.88	85.28 ± 4.13
7% tween 80	87.20 ± 0.80 ^c^	41.75 ± 3.70	85.03 ± 3.16
T. arjuna extract	90.33 ± 1.45 ^b^	41.24 ± 0.95	88.95 ± 4.72
	**Kidney function test**		
	**BUN (mg/dL)**	**Cr (mg/dL)**	
Untreated control	26.42 ± 4.32	0.66 ± 0.05	
7% tween 80	27.55 ± 0.45	0.66 ± 0.04	
T. arjuna extract	25.95 ± 2.91	0.64 ± 0.02	

Data represent mean ± SEM (*n* = 5 per group)

^a^Compared to untreated control; ^b^compared to 7% Tween 80; ^c^compared to 2000 mg/kg extract, *p* < 0.05

### Histopathological changes

**Fig. 5:**
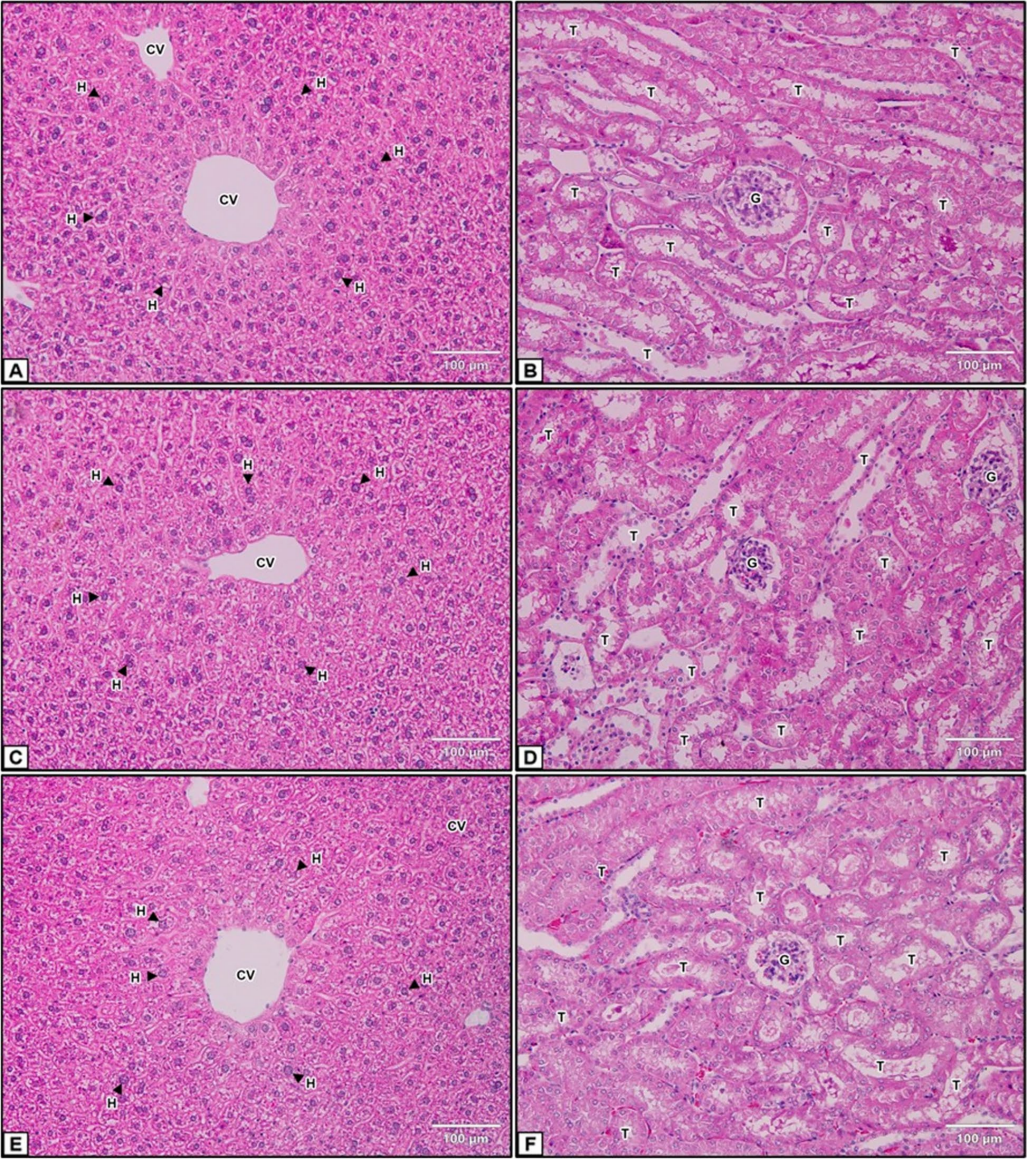
Histopathological examination of the liver and kidneys. (**A**, **B**) Untreated control group. (**C**, **D**) Negative control group. (**E**, **F**) 2000 mg/kg aqueous *T. arjuna* extract-treated group. T: tubules; G: glomerulus; CV: central vein; H: hepatocyte. Magnification: 400X

Histopathological examination revealed that the mice treated with 2000 mg/kg *T. arjuna* extract exhibited normal histopathological features in both liver and kidney tissues compared with those in the negative control group (Fig. 5). Therefore, the aqueous fruit extract of *T. arjuna* at a dose of 2000 mg/kg body weight did not have acute hepatotoxic or nephrotoxic effects.

## Discussion

Prabchompoothaweep remedy has long been used in Thai traditional medicine to relieve the common cold, hay fever, allergic rhinitis, and upper respiratory tract disease [5, 6]. The aqueous and ethanolic extracts of all plant ingredients from the Prabchompoothaweep remedy were investigated for the presence of phytochemical constituents and then for antimalarial properties against *P. falciparum* K1 strain and cytotoxicity in Vero cells.

In our in vitro study, the extracts of Prabchompoothaweep remedy and its plant ingredients were tested using enzymatic detection of the pLDH enzyme. The toxicity of the extract was next examined in Vero cells. According to the cell cytotoxicity classification, the CC_50_ value was used to define the potency of cytotoxicity. A non-toxic effect is classified as a CC_50_ value greater than 50 μg/ml [28]. The extracts exhibited varying degrees of antimalarial activity. Among the 48 crude extracts tested in the present study, the aqueous flower extract of *S. aromaticum* showed the highest antimalarial activity with the lowest IC_50_ value of 1.96 μg/ml and CC_50_ value of 134.70 μg/ml, followed by the ethanolic flower extract of *P. chaba* with IC_50_ value of 2.06 and CC_50_ value of 198.60 μg/ml followed by ethanolic rhizome extract of *Z. officinale*, aqueous fruit extract of *T. arjuna*, ethanolic fruit extract of *P. nigrum* with IC_50_ values of 3.42, 4.05 and 4.38 μg/ml and CC_50_ values of 30.73, > 54.22 and 44.36 μg/ml, respectively. Regarding *S. aromaticum*, it also known as clove. It has been reported that methanolic extract of this plant possesses slightly antimalarial effect in mice infected with *P. berghei* [32]. For *P. chaba*, piperine which is the major isolated constituent of this plant has been reported to exhibit the antimalarial effect against both chloroquine-sensitive and chloroquine-resistant *P. falciparum* clones [33]. Among the plant extracts that exhibited high antimalarial activity, the aqueous fruit extract of *T. arjuna* exhibited promising antimalarial properties, with an IC_50_ of 4.05 μg/ml. This extract possessed potent effect approximately 14.2–23.8 times compared to mildly active plants (IC_50_ ranged between 51 and 100 μg/ml). The in vitro result revealed that the CC_50_ value of the aqueous extract of *T. arjuna* was greater than 200 μg/ml, indicating that no toxic effects were present. This result is consistent with previous reports of the methanolic extract of *T. arjuna* bark exhibiting a non-cytotoxic effect on human peripheral blood mononuclear cells [34]. Therefore, the aqueous extract of *T. arjuna* was selected for further in vivo experiments.

The SI value is a crucial parameter for determining whether further work on an extract is warranted [35]. When the SI results are greater than 10, the extract is considered potentially safe in terms of cytotoxicity parameters [36]. Therefore, the aqueous *T. arjuna* extract with an SI value greater than 54.22 suppressed *P. falciparum* infection without acute toxic effects in mammalian cells. To confirm the in vitro antiplasmodial results, the antimalarial properties and toxic effects of this plant extract were further tested in an animal model.

Malaria-infected mice treated with the aqueous *T. arjuna* extract showed a significant dose-dependent decrease in the number of *Plasmodium* parasites. Furthermore, MST is an important parameter for evaluating the antimalarial activity of plant extracts. *T. arjuna* extract prolonged the survival of *P. berghei*-infected mice in a dose-dependent manner. This may be because secondary metabolites that exhibit anti-inflammatory and antioxidant functions were present and prevented the overall pathologic effect of the parasite in the infected mice [37, 38]. Since, malaria is a highly inflammatory and oxidative disease. During the blood stage of malaria infection, in response to the presence of the parasite, the host’s immune system produces proinflammatory cytokines, including IL-6, IL-8, IFN-γ, and TNF which play a pivotal role in controlling the growth of the parasite and its elimination [39]. In addition, during the blood stage of infection, the level of oxidative stress in plasma is increased, since it contributes to the elimination of invading pathogens, but also causes molecular damage in the host [40]. The potential of *T. arjuna* extract that exerts anti-inflammatory and antioxidant effects was supported by a previous report [41]. It inhibited the lipid peroxidation, maintained endogenous antioxidant enzyme activities and decreasing cytokine levels leading to decelerate the disease progression. Therefore, the antimalarial effect of the *T. arjuna* extract may be possessed by anti-inflammatory and antioxidant properties.

To confirm the safety of the extract, mice received a single dose of 2000 mg/kg aqueous *T. arjuna* extract. There were no visible signs or symptoms of toxicity or mortality in the mice. This indicated that the lethal dose of 50% was greater than 2000 mg/kg. Our study is in accordance with previous studies in which oral administration of methanolic extract of *T. arjuna* bark at various concentrations of 250–2000 mg/kg body weight did not show any adverse signs of toxicity or mortality in acute toxicity study in mice [34].

Biochemical analysis of liver and kidney functions plays an important role in evaluating the toxicological effects of xenobiotics [42, 43]. The plasma levels of ALT and ALP in mice treated with aqueous *T. arjuna* extracts were not significantly different compared with the untreated control and 7% Tween 80 groups. Regarding the kidney function test, BUN and Cr levels were not significantly different between the groups. Histopathological analysis of the liver and kidneys revealed normal features compared with those in healthy mice.

Phytochemical analysis of Prabchompoothaweep remedy showed a diversity of phytochemical constituents, including flavonoids, terpenoids, alkaloids, tannins, saponins, and coumarins. These secondary metabolites prevent the generation of free radicals and block protein synthesis in the *Plasmodium* parasite [5, 44–47]. Saponins may also modulate the immune system of infected mice [22]. Moreover, saponins are amphiphilic nature and can complex with cholesterol in biomembranes with their lipophilic moiety and bind to surface glycoproteins and glycolipids. Most terpenoids are lipophilic in nature and readily interact with the lipophilic inner core of membrane bilayers [37]. Flavonoids inhibit the influx of L-glutamine and myoinositol into *P. falciparum*-infected erythrocytes [48]. These phytochemical constituents may inhibit parasite growth and multiplication, resulting in a reduction in parasitemia and body temperature.

We found that the aqueous fruit extract of *T. arjuna* presented a group of flavonoids, terpenoids, alkaloids, tannins, and saponins. Our results are consistent with those of previous reports of the chemical constituents of *T. arjuna* [49, 50]. Secondary metabolites, particularly flavonoids, alkaloids, tannins, and saponins, are protective against *Plasmodium* parasites [44–47]. The most abundant compounds in the fruit extract of *T. arjuna* were pyrogallol, gallic acid, shikimic acid, oleamide, 5-hydroxymethylfurfural,1,1-diethoxy-ethane, quinic acid, and furfural. The antimalarial activity of this extract may be attributed to the synergistic effects of these compounds. Interestingly, the identified compounds from the fruit extract of *T. arjuna* including pyrogallol, gallic acid, shikimic acid, cinnamic acid, and quinic acid are interrelated via biosynthesis pathway [29–31]. Since, most non-volatiles will decompose at between 400 and 1000 °C [51]. Therefore, the identified compounds which non-volatiles cannot be vaporized and decomposed easily. In addition, in this study, the injector temperature of GC-MS was set at a constant of 250 °C, and the maximum temperature of the oven was set at 300 °C. So, this thermal condition inapplicable for decomposition process.

The bioactivities of the two major compounds, shikimic acid and 5-hydroxymethylfurfural, further explains why the *T. arjuna* extracts significantly enhanced the survival in mice. For instance, shikimic acid a key intermediate in the biosynthesis of aromatic compounds, exerts antibacterial, anti-inflammatory, analgesic, antioxidant, antithrombotic, and antibacterial activities [52]; 5-hydroxymethylfurfural is a furan-containing aldehyde present in sacchariferous foods (fruit juices and dried fruits), *Codonopsis pilosula* and garlic exerts antioxidant, anti-inflammatory, anti-proliferative, and cardioprotective effects [53, 54]. For, gallic acid or 3, 4, 5-trihydroxybenzoic acid, it has been reported to exhibit various pharmacological properties, including antibacterial, antiviral and antitumor activities [55]. In addition, this compound isolated from *Alectryon serratus* leaves possessed antiplasmodial activity against chloroquine-sensitive 3D7 strain of *P. falciparum* with IC_50_ value of 0.0722 μM [56].

Regarding pyrogallol or 1, 2, 3-benzenetriol which is an organic phenol compound that exists naturally in many plants such as *Terminalia chebula*, *Myriophyllum spicatum* and *Diospyros chamaethamnus.* It possesses antibacterial, antipsoriatic, antifungal properties, and revealed antimalarial activity against *P. falciparum* chloroquine-sensitive strain [57, 58]. The potential of pyrogallol to exert antimalarial activity was supported by its property that it is autoxidised rapidly in solutions ranging from pH 3.5–4.5 and generates various free radicals such as peroxide nitrite, hydrogen peroxide, and hydroxyradical. These free radicals may enable the inhibition of parasite growth [57].

## Conclusions

A total of 10 plants from 23 medicinal plant ingredients of Prabchompoothaweep remedy showed high in vitro antimalarial activity. Among these, the aqueous fruit extract of *T. arjuna* possessed potent effect approximately 14.2–23.8 times compared to mildly active plants (IC_50_ ranged between 51 and 100 μg/ml). This extract exerts antimalarial activity against *Plasmodium* parasites found in humans (*P. falciparum* K1 strain) and mice (*P. berghei* ANKA strain). Acute toxicity studies revealed that the aqueous fruit extract of *T. arjuna* did not present any lethality or adverse effects up to a dose of 2000 mg/kg body weight. These results suggest that *T. Arjuna* extract has antimalarial activity that could be a promising starting point for the study of the antimalarial drug.

Therefore, further studies on the aqueous extract of *T. arjuna* should focus on its phytochemical contents to identify their bioactive constituents and mechanisms of parasite inhibition including studies on a synergistic effect of the combinations of the abundant phytochemicals identified in *T. arjuna* extracts.

## Data Availability

The data used to support the findings of this study have been included in this article. Additional files are available from the corresponding authors upon request.

## Abbreviations

pLDH: *Plasmodium* lactate dehydrogenase enzyme
GC-MS: Chromatography-mass spectrometry
IC_50_: Half-maximal inhibitory concentration
CC_50_: 50% cytotoxic concentration
RBCs: Red blood cells
DMSO: Dimethyl sulfoxide
MTT: 3-(4, 5-dimethylthiazol-2-yl)-2, 5-diphenyltetrazolium bromide
SI: Selectivity index
NIST: National Institute of Standards and Technology
ICR mice: Institute of Cancer Research mice
MST: Mean survival time
ALT: Alanine aminotransferase
AST: Aspartate aminotransferase
ALP: Alkaline phosphatase
BUN: Blood urea nitrogen
Cr: Creatinine
SEM: Standard error of the mean
FL: Flavonoids
TN: Terpenoids
AL: Alkaloids
TA: Tannins
AN: Anthraquinones
CG: Cardiac glycosides
SA: Saponins
CM: Coumarins
